# Overnight Continuous Saline Bladder Irrigation After *En Bloc* Resection of Bladder Tumor Does Not Improve Oncological Outcomes in Patients Who Have Received Intravesical Chemotherapy

**DOI:** 10.3389/fonc.2021.638065

**Published:** 2021-03-10

**Authors:** Yongjun Yang, Chao Liu, Xiaoting Yan, Jiawei Li, Xiaofeng Yang

**Affiliations:** ^1^ First Clinical Medical College, Shanxi Medical University, Taiyuan, China; ^2^ Department of Urology, First Hospital of Shanxi Medical University, Taiyuan, China

**Keywords:** non-muscle invasive bladder cancer, *en bloc* resection, continuous saline bladder irrigation, intravesical chemotherapy, recurrence

## Abstract

**Objective:**

To evaluate the safety and efficacy of overnight continuous saline bladder irrigation (CSBI) for patients who have received thulium laser *en bloc* resection of bladder tumor (TmLRBT) combined with immediate intravesical chemotherapy previously.

**Methods:**

From October 2014 to June 2018, 235 patients with newly diagnosed non-muscle invasive bladder cancer (NMIBC) were included in this retrospective study. All patients received intravesical instillation of pirarubicin immediately after TmLRBT. The patients were divided into two groups according to the duration of postoperative bladder irrigation with normal saline. After immediate intravesical chemotherapy, patients in group 1 received overnight CSBI, while patients in group 2 did not receive overnight CSBI. Data on the time of initial tumor recurrence, recurrence-free survival (RFS) and progression-free survival (PFS) rates, and perioperative complications were collected and analyzed.

**Results:**

Of 235 included patients (129 in group 1 and 106 in group 2), the median follow-up periods were 42 and 38 months, respectively. There were no significant differences in patients’ baseline characteristics between the two groups. The RFS rates of patients in group 1 were 90.7, 82.7, and 76.8% at the end of the first, third, and fifth years, while the corresponding RFS rates of patients in group 2 were 87.7, 78.9, and 73.3%, respectively. Four patients in group 1 and five patients in group 2 experienced tumor progression. No significant differences between the two groups were observed in the time of initial tumor recurrence, RFS, and PFS rates. Only Grade I complications occurred in the two groups, and no significant difference was reached between the two groups.

**Conclusions:**

For patients with NMIBC who have previously received TmLRBT combined with immediate intravesical chemotherapy, overnight CSBI may not improve oncological outcomes and reduce perioperative complications.

## Introduction

Bladder cancer (BC) is the tenth most common cancer disease worldwide with 474,000 new incident cases and 197,000 deaths annually, and it is also the second most common malignant disease of the urinary system after prostate cancer (1). Approximately 75% of newly diagnosed BC presents as malignant lesions confined to the mucosa or submucosa, which are collectively referred to as non-muscle invasive bladder cancer (NMIBC) (2). For these patients, transurethral resection of bladder tumor (TURBT) combined with individualized intravesical chemotherapy or immunotherapy that is tailored to tumor risk stratification is recommended as the routine treatment model by the major international guidelines (2–5). However, piecemeal resection of tumor tissue in conventional TURBT results in exfoliated tumor cell dissemination and seeding, which goes against the recognized principle of oncological surgery and partly contributes to increasing the out of field recurrence (6, 7).

Initially, after transurethral tumor resection, continuous saline bladder irrigation (CSBI) was used to prevent the formation of blood clots and achieve excellent hemostasis. Meanwhile, in theory, CSBI can flush out exfoliated tumor cells effectively and prevent them from implanting in the bladder mucosa, thereby reducing the risk of tumor recurrence after conventional resection (8). However, CSBI has no therapeutic effect on the residual tumors at the initial resection site, so it is necessary to perform high-quality and complete tumor resection to make sure that the tumor specimens contain the lamina propria and superficial muscular layer (9).

In the past decade, *en bloc* resection of bladder tumor (ERBT) served as a valuable alternative technique that has obtained increasing interest among urologists worldwide (10). As a “no touch” surgical technique for the treatment of NMIBC, ERBT shows the potential to minimize the number of exfoliated tumor cells and reduce the risk of tumor cell reimplantation. The use of thulium laser as the energy source for ERBT does not generate high-frequency current and has excellent hemostatic effect. Therefore, the incidence of perioperative complications, such as obturator nerve reflex (ONR), bladder perforation (BP), and acute bleeding will be reduced (11). Then, we tested the hypothesis that overnight CSBI has little effect on improving oncological outcomes and reducing the incidence of perioperative complications for patients with NMIBC who have received thulium laser *en bloc* resection of bladder tumor (TmLRBT) combined with immediate intravesical chemotherapy.

## Patients and Methods

### Patients

From October 2014 to June 2018, all patients with newly diagnosed NMIBC, who underwent a TmLRBT combined with immediate intravesical instillation of pirarubicin were retrospectively included. Cystoscopy, ultrasonography, intravenous pyelography, and computed tomography were performed to select the appropriate patients before TmLRBT. The inclusion criteria were as follows: 1) patients who underwent TmLRBT successfully without switching to conventional TURBT; 2) patients were diagnosed with NMIBC for the first time; 3) detrusor muscle was contained in the tumor specimens. The exclusion criteria were as follows: 1) preoperative examinations revealed distant metastasis or pelvic lymph node metastasis; 2) carcinoma *in situ* (CIS) and upper urinary tract neoplasms were accompanied; 3) the intact tumor specimens were cut into two or three parts longitudinally in the bladder before being retrieved; 4) histopathological analysis of the tumor specimens showed that the muscle layer of the bladder was invaded. This study protocol was approved by the Ethics Committee of the First Hospital of Shanxi Medical University. All patients were informed and agreed to participate in the study.

### Surgical Procedure

The surgical procedure of white light cystoscopy-assisted TmLRBT was performed by the same urologist with rich experience in endoscopy. All patients were placed in a lithotomy position under combined spinal and epidural anesthesia. Sterile normal saline was used for continuous bladder irrigation, and the Revolix™ thulium laser system (LISA Laser Products, Lindau-Katlenburg, Germany) was used as the energy source during TmLRBT. Firstly, the urologist examined the entire bladder mucosa thoroughly and recorded the tumor location, number, size, and appearance. Then, a circular mucosal incision was made at a safe distance of 5–10 mm from the base of the tumor tissue, and the visible blood vessels near the tumor tissue were coagulated and blocked at the same time. At the circular incision line, the urologist first made a vertical incision from the bladder mucosa to the deep muscular layer and then removed the whole tumor tissue by vapor resection with the thulium laser and blunt dissection with the tip of the resectoscope. In order to minimize the cautery artifacts of the tumor specimens, laser vapor resection was mainly used to cut off the muscle fibers around the tumor. Finally, the intact tumor specimens were retrieved with an Ellick’s evacuator *via* out sheath of the resectoscope. For the larger-size tumor specimens, an additional medical device, such as laparoscopic forceps, was required to complete the work.

### Intravesical Chemotherapy

Immediate intravesical chemotherapy with 30 mg pirarubicin was administered within 6 h after TmLRBT. The duration of pirarubicin in the bladder was 1 h, and then patients in group 1 received overnight CSBI (2,000 ml/h for first 1 h, then 1,000 ml/h for 3 h, and then 250 ml/h for 12 to16 h) after intravesical chemotherapy, while patients in group 2 did not receive overnight CSBI. After discharge from the hospital, patients with intermediate- and high-risk NMIBC received maintenance intravesical chemotherapy with pirarubicin for one year. The detailed scheme of intravesical instillation of pirarubicin was once a week for 8 weeks, followed by monthly intravesical chemotherapy to 12 months. Based on the European Association of Urology (EAU) guidelines, a second transurethral resection was performed in patients with T1 and/or high-grade bladder tumor (12).

### Follow-Up Strategies

Patients with low-risk NMIBC were followed up every 3 months in the first year, every 6 months in the second year, and then once a year. For patients with intermediate- and high-risk NMIBC, the follow-up strategy was every 3 months for the first two years, then every 6 months until the fifth year, and then once a year. Routine examination items included ultrasonography, urine cytology, and cystoscopy.

The time of initial tumor recurrence was defined as the time interval between TmLRBT and the date of initial tumor recurrence. When cystoscopy showed space-occupying lesions in the bladder, tumor recurrence should be considered. Tumor progression was defined as an increase in pathological stage. All recurrent and progressive tumor lesions were further confirmed by histopathological assessment. Histological grade and pathological stage of the tumor specimens were evaluated according to the 2004 World Health Organization (WHO) grading system and the 2009 version of TNM staging system, respectively.

### Statistical Analysis

Continuous data were expressed as median and tested using unpaired t-test. Qualitative data were described as numbers and percentages and compared through Chi-square test or Fisher’s exact test. The patients’ baseline characteristics and CSBI were included in multivariate Cox proportional hazard models to determine which variables correlate to recurrence-free survival (RFS). The RFS and progression-free survival (PFS) curves were estimated by the Kaplan–Meier method, and the difference between survival curves was analyzed by the log-rank test. Statistical analysis was performed using GraphPad Prism statistical software package, version 7.0 for Windows and IBM SPSS statistics, version 19.0 for Windows. All tests were two-sided, and statistical significance was reached when the *P* value <0.05.

## Results

The study population included 254 patients with newly diagnosed NMIBC who received TmLRBT. After reviewing the patients’ clinical data, 19 cases were excluded due to the following reasons: six cases were accompanied with CIS, the intact tumor specimens of eight cases were cut into pieces in the bladder before being retrieved, and five cases switched to conventional TURBT during surgery. The remaining 235 patients were divided into two groups according to the duration of postoperative bladder irrigation with normal saline. Patients in group 1 (n = 129) received overnight CSBI after intravesical chemotherapy, while patients in group 2 (n = 106) did not receive overnight CSBI. The patients’ baseline characteristics in the two groups were compared in terms of age, gender, smoking history, tumor diameter, number, grade and stage, and EAU risk stratification. The results showed that no significant differences existed between the two groups (**Table 1**).

**Table 1:** Baseline characteristics of patients in the two groups.

Characteristics	Group 1 (n = 129)	Group 2 (n = 106)	*P*
Age (year)	66 (24–84)	65.5 (38–82)	0.62
Gender			
Male	103 (79.84%)	83 (78.30%)	0.87
Female	26 (20.16%)	23 (21.70%)	
Smoking history			0.86
Current	58 (44.96%)	44 (41.51%)	
Prior	36 (27.91%)	32 (30.19%)	
Never	35 (27.13%)	30 (28.30%)	
Tumor diameter (cm)			0.89
1.0–2.0 cm	90 (69.77%)	75 (70.75%)	
2.0–3.0 cm	39 (30.23%)	31 (29.25%)	
Tumor number			0.68
Single	84 (65.12%)	72 (67.92%)	
Multiple	45 (34.88%)	34 (32.08%)	
Grade (WHO 2004)			0.51
Low-grade	76 (58.91%)	57 (53.77%)	
High-grade	53 (41.09%)	49 (46.23%)	
T stage			0.87
Ta	106 (82.17%)	86 (81.13%)	
T1	23 (17.83%)	20 (18.87%)	
EAU risk stratification			0.93
Low-risk	37 (28.68%)	28 (26.42%)	
Intermediate-risk	62 (48.06%)	53 (50.00%)	
High-risk	30 (23.26%)	25 (23.58%)	

WHO, World Health Organization; EAU, European Association of Urology.

**Figure 1:**
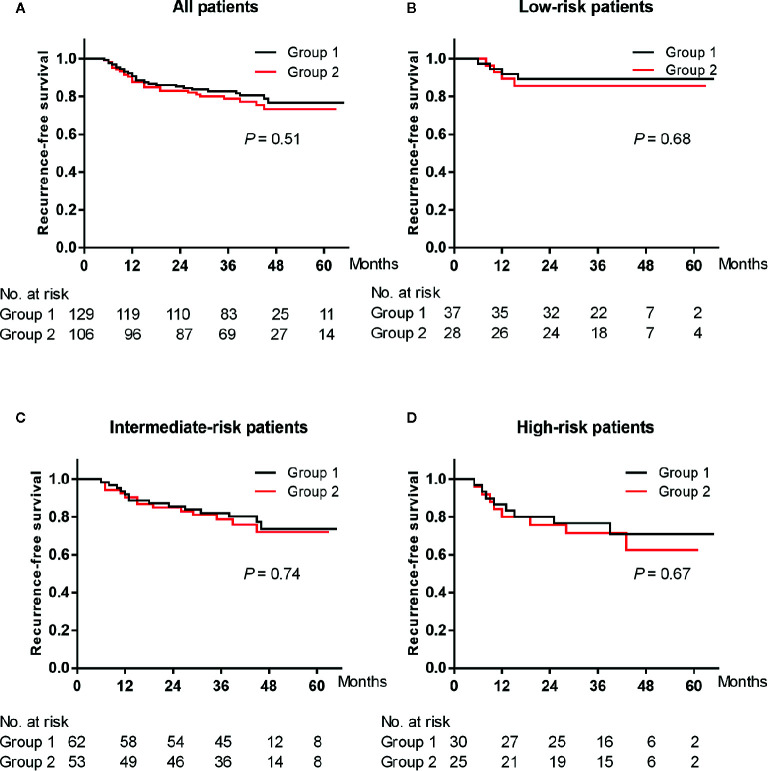
Kaplan-Meier plot of the recurrence-free survival rates of all **(A)** and low-risk **(B)**, intermediate-risk **(C)** and high-risk **(D)** patients treated with overnight continuous saline bladder irrigation or not after thulium laser en bloc resection of bladder tumor combined with immediate intravesical chemotherapy.

Median follow-up period was 42 months (range 5–65 months) in group 1 and 38 months (range 5–63 months) in group 2. Twenty-six (20.2%) cases in group 1 and twenty-five (23.6%) cases in group 2 developed tumor recurrence during the follow-up period. In group 1, the RFS rates were 90.7, 82.7, and 76.8% at the end of the first, third, and fifth years. In group 2, the RFS rates were 87.7, 78.9, and 73.3% at the end of the first, third, and fifth years, respectively. The Kaplan–Meier curve of the RFS rate of all patients showed no significant difference between the two groups (log-rank test: *P* = 0.51) (**Figure 1A**). The RFS rates of patients with low-, intermediate- and high-risk NMIBC also did not reach a significant difference between the two groups (log-rank test: *P* = 0.68, 0.74 and 0.67, respectively) (**Figures 1B–D**). In multivariate analysis, overnight CSBI was not an independent predictor of RFS (HR 0.76, *P* = 0.357) (**Table 2**).

**Table 2:** Multivariate Cox proportional hazard analyses of recurrence-free survival in all patients.

Characteristics	RFS multivariate	
	HR (95% CI)	*P*
Age (year)	0.98 (0.96–1.01)	0.145
Gender (male)	1.29 (0.53–3.15)	0.580
Smoking (yes)	1.35 (0.65–2.80)	0.416
Diameter (2.0–3.0 cm)	1.69 (0.95–3.01)	0.077
Number (multiple)	2.55 (1.36–4.78)	0.003
Grade (high)	1.32 (0.73–2.39)	0.367
Stage (T1)	1.28 (0.54–3.03)	0.577
risk stratification (high)	3.85 (1.54–9.62)	0.004
CSBI (yes)	0.76 (0.43–1.36)	0.357

RFS, recurrence-free survival; CSBI, continuous saline bladder irrigation; HR, hazard ratio; CI, confidence interval.

**Figure 2:**
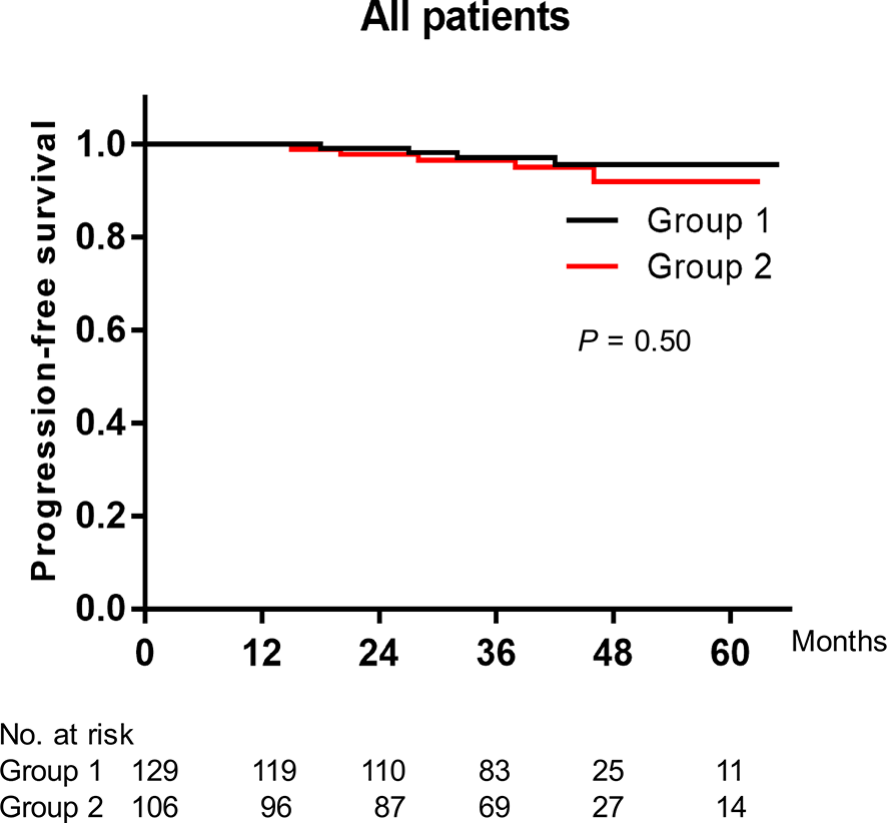
Kaplan-Meier plot of the progression-free survival rates of all patients treated with overnight continuous saline bladder irrigation or not after thulium laser en bloc resection of bladder tumor combined with immediate intravesical chemotherapy.

The median period of initial tumor recurrence was 13 months in group 1 and 12 months in group 2, and no significant difference was observed between the two groups (unpaired t-test: *P* = 0.96). Four (3.1%) cases in group 1 and five (4.7%) cases in group 2 developed tumor progression during the follow-up period. There was no significant difference in PFS rates between the two groups (log-rank test: *P* = 0.50) (**Figure 2**).

Perioperative complications were recorded in all 235 patients. Based on the modified Clavien classification system for surgical complications (13), only Grade I complications happened in the two groups. The incidence of Grade I complications were 9.3% (12/129) and 12.3% (13/106) in group 1 and group 2, respectively. Although the incidence of complications in group 1 was less, there was no significant difference between the two groups (Fisher’s exact test: *P* = 0.53). No patient experienced ONR and BP during operation (**Table 3**).

**Table 3:** Perioperative complications.

	Group 1 (n = 129)	Group 2 (n = 106)	*P*
Obturator nerve reflex	0	0	–
Bladder perforation	0	0	–
Complications			0.53
Grade I	12 (9.30%)	13 (12.26%)	
≥Grade II	0	0	

## Discussion

The well-known principle of oncological surgery is to resect the tumor tissue in one piece with negative surgical margins and prevent iatrogenic tumor cells scattering and local implantation. However, during conventional TURBT, the tumor tissue is resected piece by piece from the exophytic part of the tumor to the superficial muscular layer by a wire loop. The integrity of the tumor tissue is destroyed, and tumor cells are dispersed, which may increase the risk of exfoliated tumor cell dissemination and implantation. Compared with conventional TURBT, ERBT adheres to the basic principle of cancer surgery and provides pathologists with an intact tumor specimen for accurate histopathological analysis (14, 15). Meanwhile, as a “no touch” technique for the treatment of NMIBC, ERBT shows the potential to minimize the number of exfoliated tumor cells, and then may reduce the risk of tumor cell reimplantation.

In several studies on ERBT, the duration of CSBI after operation was recorded. However, there were huge differences between the relevant data. Xu et al. analyzed the safety and efficacy of 1.9 µm Vela laser ERBT for the treatment of NMIBC in a retrospective study, and the mean duration of postoperative CSBI was 29.1 h (16). A European multicenter prospective study was conducted to compare the safety and efficacy of ERBT using different energy sources. After tumor resection with electrical current and laser energy, the mean periods of bladder irrigation were 0.76 and 0.63 days, respectively (17). Li et al. explored the safety and efficacy of TmLRBT for the treatment of NMIBC in a retrospective study, and the median duration of postoperative bladder irrigation with normal saline was 6.33 h (18). The monopolar current, initially used in conventional TURBT, was also applied as the energy source for *en bloc* resection. And the median time of bladder irrigation after ERBT was 1.16 days (19). While in a prospective study, no patient required bladder irrigation after 980 nm laser ERBT (20). Therefore, we question how to choose the best duration of bladder irrigation after ERBT. In this retrospective study, after TmLRBT combined with immediate intravesical chemotherapy, there were no significant differences in the time of initial tumor recurrence, RFS, and PFS rates between the patients who received overnight CSBI or not. Hence, when excellent hemostasis effect is obtained during ERBT, it is safe not to perform overnight CSBI after surgery.

The function of CSBI is to achieve excellent hemostasis and remove blood clots in the bladder. Meanwhile, continuous bladder irrigation after tumor resection can wash away exfoliated tumor cells and reduce the risk of tumor cell reimplantation in the injured bladder mucosa (21). However, CSBI has no therapeutic effect on the residual tumors at the initial resection site. Then, in order to achieve the effect of postoperative bladder irrigation to reduce tumor recurrence, the prerequisite is high quality and complete tumor resection. Unlike ERBT using electrical current as the energy source, no high-frequency current is generated during TmLRBT. Combined with more precise and controllable procedure of tumor resection, ONR and BP can be avoided (22). After surgery, patients can receive intravesical chemotherapy immediately without being restricted by perioperative complications, such as acute bleeding and BP. Thulium laser, as a diode pumped solid-state laser, works in continuous fashion at the wavelength of 2,013 nm and the penetration depth of 250 μm. Compared with pulsed holmium laser, ERBT using thulium laser as the energy source makes tumor resection more precise and controllable, and the hemostatic effect is excellent (23). Due to its excellent hemostatic effect, bladder irrigation may not be required after surgery to prevent the formation of blood clots in the bladder. In this trial, the results indicated that there were no significant differences in oncological outcomes and perioperative complications between patients who received overnight CSBI or not. Therefore, for well-selected patients with newly diagnosed NMIBC, TmLRBT combined with immediate intravesical chemotherapy can be performed in the day-surgery unit without the need of overnight CSBI. This is a better mode of allocating medical resources, which alleviates the logistical pressure caused by the expansion of the waiting number to a certain extent. It is also a process of reducing health-care costs and has a positive impact on medical expenses (24).

The main limitation of the trial is its retrospective design and relatively small patient population. However, we believe that our preliminary findings are very meaningful for urologists and patients with NMIBC. For patients with newly diagnosed NMIBC, urologists can choose to perform TmLRBT as day-surgery operation on them to shorten the waiting time outside the hospital, and then reduce the patient’s nervousness related to waiting outside the hospital (25). Further prospective randomized controlled trials with more patients are needed to confirm our results. Second, thulium is a less commonly used laser energy source in transurethral tumor resection, and ERBT is less commonly performed than conventional TURBT. Due to the excellent hemostatic effect of thulium and the theoretical benefits of ERBT, TmLRBT may gain more and more interest among urologists. Third, although pirarubicin is not as widely used in intravesical chemotherapy as gemcitabine or mitomycin C (MMC), in a systematic review, indirect comparisons could not detect any differences in efficacy between MMC and pirarubicin (26).

## Conclusions

For patients with newly diagnosed NMIBC, after TmLRBT combined with immediate intravesical chemotherapy, overnight CSBI cannot improve oncological outcomes and reduce the incidence of perioperative complications. Therefore, TmLRBT may be performed as day-surgery operation for well-selected patients.

## Data Availability Statement

The original contributions presented in the study are included in the article/supplementary material. Further inquiries can be directed to the corresponding author.

## Ethics Statement

The study was approved by the Ethics Committee of the First Affiliated Hospital of Shanxi Medical University (2011033/2011) and conducted according to the principles of the Declaration of Helsinki.

## Author Contributions

YY designed the clinical study, collected and analyzed the clinical data, and wrote the manuscript. CL analyzed and interpreted the patient data. XTY and JL collected and analyzed the clinical data. XFY designed the study, supervised the research, contributed to the experimental discussion, and reviewed the manuscript. All authors contributed to the article and approved the submitted version.

## Funding

This research was supported by the National Natural Science Foundation of China Grants (81172444 to XFY).

## Conflict of Interest

The authors declare that the research was conducted in the absence of any commercial or financial relationships that could be construed as a potential conflict of interest.

## Abbreviations

BC, Bladder cancer; NMIBC, Non-muscle invasive bladder cancer; TURBT, Transurethral resection of bladder tumor; CSBI, Continuous saline bladder irrigation; ERBT, *En bloc* resection of bladder tumor; ONR, Obturator nerve reflex; BP, Bladder perforation; TmLRBT, Thulium laser *en bloc* resection of bladder tumor; CIS, carcinoma *in situ*; EAU, European Association of Urology; WHO, World Health Organization; RFS, recurrence-free survival; PFS, progression-free survival; MMC: mitomycin C.

## References

[1] Global Burden of Disease Cancer Collaboration Fitzmaurice C Abate D Abbasi N Abbastabar H Abd-Allah F. Global, Regional, and National Cancer Incidence, Mortality, Years of Life Lost, Years Lived With Disability, and Disability-Adjusted Life-Years for 29 Cancer Groups, 1990 to 2017: A Systematic Analysis for the Global Burden of Disease Study. JAMA Oncol (2019) 5:1749–68. 10.1001/jamaoncol.2019.2996 PMC6777271 31560378

[2] Babjuk M Burger M Compérat EM Gontero P Mostafid AH Palou J. European Association of Urology Guidelines on Non-muscle-invasive Bladder Cancer (TaT1 and Carcinoma In Situ) - 2019 Update. Eur Urol (2019) 76:639–57. 10.1016/j.eururo.2019.08.016 31443960

[3] Flaig TW Spiess PE Agarwal N Bangs R Boorjian SA Buyyounouski MK. Bladder Cancer, Version 3.2020, NCCN Clinical Practice Guidelines in Oncology. J Natl Compr Canc Netw (2020) 18:329–54. 10.6004/jnccn.2020.0011 32135513

[4] Kassouf W Traboulsi SL Kulkarni GS Breau RH Zlotta A Fairey A. CUA guidelines on the management of non-muscle invasive bladder cancer. Can Urol Assoc J (2015) 9:E690–704. 10.5489/cuaj.3320 PMC4662433 26664503

[5] Chang SS Boorjian SA Chou R Clark PE Daneshmand S Konety BR. Diagnosis and Treatment of Non-Muscle Invasive Bladder Cancer: AUA/SUO Guideline. J Urol (2016) 196:1021–9. 10.1016/j.juro.2016.06.049 27317986

[6] Babjuk M Burger M Compérat EM Gontero P Mostafid HA Palou J. Indication for a Single Postoperative Instillation of Chemotherapy in Non-muscle-invasive Bladder Cancer: What Factors Should Be Considered? Eur Urol Focus (2018) 4:525–8. 10.1016/j.euf.2018.07.023 30061076

[7] Bălan GX Geavlete PA Georgescu DA Ene CV Bulai CA Păunescu MA. Bipolar en bloc tumor resection versus standard monopolar TURBT - which is the best way to go in non-invasive bladder cancer? Rom J Morphol Embryol (2018) 59:773–80. 30534816

[8] Zhou Z Zhao S Lu Y Wu J Li Y Gao Z. Meta-analysis of efficacy and safety of continuous saline bladder irrigation compared with intravesical chemotherapy after transurethral resection of bladder tumors. World J Urol (2019) 37:1075–84. 10.1007/s00345-019-02628-7 30612154

[9] Kramer MW Altieri V Hurle R Lusuardi L Merseburger AS Rassweiler J. Current Evidence of Transurethral En-bloc Resection of Nonmuscle Invasive Bladder Cancer. Eur Urol Focus (2017) 3:567–76. 10.1016/j.euf.2016.12.004 28753835

[10] Teoh JY MacLennan S Chan VW Miki J Lee HY Chiong E. An International Collaborative Consensus Statement on En Bloc Resection of Bladder Tumour Incorporating Two Systematic Reviews, a Two-round Delphi Survey, and a Consensus Meeting. Eur Urol (2020) 78:546–69. 10.1016/j.eururo.2020.04.059 32389447

[11] Territo A Bevilacqua G Meneghetti I Mercadé A Breda A. En bloc resection of bladder tumors: indications, techniques, and future directions. Curr Opin Urol (2020) 30:421–7. 10.1097/MOU.0000000000000737 32205806

[12] Babjuk M Burger M Zigeuner R Shariat SF van Rhijn BW Compérat E. EAU guidelines on non-muscle-invasive urothelial carcinoma of the bladder: update 2013. Eur Urol (2013) 64:639–53. 10.1016/j.eururo.2013.06.003 23827737

[13] De Nunzio C Franco G Cindolo L Autorino R Cicione A Perdonà S. Transuretral resection of the bladder (TURB): analysis of complications using a modified Clavien system in an Italian real life cohort. Eur J Surg Oncol (2014) 40:90–5. 10.1016/j.ejso.2013.11.003 24284200

[14] Xu S Tan S Wu T Gu J Xu L Che X. The value of transurethral thulium laser en bloc resection combined with a single immediate postoperative intravesical instillation of pirarubicin in primary non-muscle-invasive bladder cancer. Lasers Med Sci (2020) 35:1695–701. 10.1007/s10103-020-02960-0 31970565

[15] Liang H Yang T Wu K He D Fan J. En bloc resection improves the identification of muscularis mucosae in non-muscle invasive bladder cancer. World J Urol (2019) 37:2677–82. 10.1007/s00345-019-02672-3 30830276

[16] Xu H Ma J Chen Z Yang J Yuan H Wang T. Safety and Efficacy of En Bloc Transurethral Resection With 1.9 µm Vela Laser for Treatment of Non-Muscle-invasive Bladder Cancer. Urology (2018) 113:246–50. 10.1016/j.urology.2017.11.030 29198850

[17] Kramer MW Rassweiler JJ Klein J Martov A Baykov N Lusuardi L. En bloc resection of urothelium carcinoma of the bladder (EBRUC): A European multicenter study to compare safety, efficacy, and outcome of laser and electrical en bloc transurethral resection of bladder tumor. World J Urol (2015) 33:1937–43. 10.1007/s00345-015-1568-6 25910478

[18] Li K Xu Y Tan M Xia S Xu Z Xu D. A retrospective comparison of thulium laser en bloc resection of bladder tumor and plasmakinetic transurethral resection of bladder tumor in primary non-muscle invasive bladder cancer. Lasers Med Sci (2019) 34:85–92. 10.1007/s10103-018-2604-8 30171441

[19] Zhang KY Xing JC Li W Wu Z Chen B Bai DY. A novel transurethral resection technique for superficial bladder tumor: retrograde en bloc resection. World J Surg Oncol (2017) 15:125. 10.1186/s12957-017-1192-6 28683751 PMC5501371

[20] Tao W Sun C Yao Q Fu K Shan Y Zhang Y. The clinical study of en bloc transurethral resection with 980 nm laser for treatment of primary non-muscle invasive bladder cancer. J Xray Sci Technol (2020) 28:563–71. 10.3233/XST-190616 32224536

[21] Onishi T Sugino Y Shibahara T Masui S Yabana T Sasaki T. Randomized controlled study of the efficacy and safety of continuous saline bladder irrigation after transurethral resection for the treatment of non-muscle-invasive bladder cancer. BJU Int (2017) 119:276–82. 10.1111/bju.13599 27444991

[22] Wang W Liu H Xia S. Thulium laser treatment for bladder cancer. Asian J Urol (2016) 3:130–3. 10.1016/j.ajur.2016.05.002 PMC5730823 29264180

[23] Enikeev D Shariat SF Taratkin M Glybochko P. The changing role of lasers in urologic surgery. Curr Opin Urol (2020) 30:24–9. 10.1097/MOU.0000000000000695 31724998

[24] Sloan FA Yashkin AP Akushevich I Inman BA. The Cost to Medicare of Bladder Cancer Care. Eur Urol Oncol (2019) 3:515–22. 10.1016/j.euo.2019.01.015 31412015

[25] Tan WS Teo CH Chan D Ang KM Heinrich M Feber A. Exploring patients’ experience and perception of being diagnosed with bladder cancer: a mixed-methods approach. BJU Int (2020) 125:669–78. 10.1111/bju.15008 PMC7318301 31975539

[26] Sylvester RJ Oosterlinck W Holmang S Sydes MR Birtle A Gudjonsson S. Systematic Review and Individual Patient Data Meta-analysis of Randomized Trials Comparing a Single Immediate Instillation of Chemotherapy After Transurethral Resection with Transurethral Resection Alone in Patients with Stage pTa-pT1 Urothelial Carcinoma of the Bladder: Which Patients Benefit from the Instillation? Eur Urol (2016) 69:231–44. 10.1016/j.eururo.2015.05.050 26091833

